# Antibacterial Effect of Silver Nanoparticles Is Stronger If the Production Host and the Targeted Pathogen Are Closely Related

**DOI:** 10.3390/biomedicines10030628

**Published:** 2022-03-08

**Authors:** Priyanka Singh, Ivan Mijakovic

**Affiliations:** 1 The Novo Nordisk Foundation, Center for Biosustainability, Technical University of Denmark, DK-2800 Kogens Lyngby, Denmark; 2 Systems and Synthetic Biology Division, Department of Biology and Biological Engineering, Chalmers University of Technology, SE-412 96 Gothenburg, Sweden

**Keywords:** silver nanoparticles, stability, strong antibacterial activity, corona, reusability

## Abstract

Microbial resistance to antibiotics is one of the key challenges that lead to the search for alternate antimicrobial treatment approaches. Silver nanoparticles (AgNPs) are well known for their antimicrobial effects against a wide variety of drug-resistant microorganisms. AgNPs can be synthesized using microbial hosts, using a green and economical synthesis route, which produces extremely stable and highly active nanoparticles. Such green AgNPs are coated with a biological coating often referred to as a corona, originating from the production microorganism. In this study, we asked whether the composition of the biological corona might influence the antimicrobial activity of green AgNPs. To investigate this, we produced AgNPs in *Pseudomonas putida* KT2440 and *Escherichia coli* K12 MG1655, and tested them against pathogen species from the corresponding genera. AgNPs exhibited a size range of 15–40 nm for *P. putida* and 30–70 nm for *E. coli*, and both types of nanoparticles were surrounded by a thick biological corona layer, providing extreme stability. The nanoparticles remained stable over long periods and exhibited negative zeta potential values. P-AgNPs (obtained from *P. putida*) were tested against pathogenic *Pseudomonas aeruginosa* PAO1, and E-AgNPs (obtained from *E. coli*) were tested against pathogenic *Escherichia coli* UTI 89. Antimicrobial studies were conducted by Minimum bactericidal concentration (MBC), live/dead staining and SEM analysis. MBC of P-AgNPs against *P. aeruginosa* was 1 μg/mL, and MBC of E-AgNPs against *E. coli* UTI 89 was 8 μg/mL. In both cases, the MBC values were superior to those of green AgNPs produced in organisms unrelated to the target pathogens, available in the literature. Our results suggest that NPs produced in microorganisms closely related to the target pathogen may be more effective, indicating that the composition of the biological corona may play a crucial role in the antimicrobial mechanism of AgNPs.

## 1. Introduction

Infectious diseases caused by drug-resistant microorganisms are a serious public health concern. To address these infections, scientists are continuously developing novel antimicrobial agents. Silver nanoparticles (AgNPs) are very popular for their antimicrobial activity against multidrug-resistant pathogens [1]. For a long time, AgNPs have been applied as broad-spectrum antimicrobial agents against many pathogenic and non-pathogenic microorganisms in various industrial applications like food packaging and textiles industries, etc. [2]. Especially, AgNPs showed wide applications in the healthcare domain, including wound infection treatment, tumor targeting, drug delivery, sensors, etc. [3,4]. The major advantage of using AgNPs for antimicrobial purposes is their higher toxicity against microorganisms with high permeability at low dosage due to the size and shape of nanoparticles (NPs) [5]. The remarkable features of nanosized materials are attributed primarily to their large surface area to volume ratio [6]. Conventionally, various physical and chemical methods were employed for AgNPs production, involving chemical reductions, ultra-sonication, radiolysis, microwave-assisted synthesis, electrospinning, sol-gel method, or radiations-based methods, which further use various capping agents to prevent aggregation. Although chemical reduction methods are easy to control and effectively achieve high monodispersity and shape specificity, they are relatively expensive and often involve toxic chemicals that have unforeseen consequences for the environment and human health [7]. In some cases, the process involves a prolonged synthesis and the need for harmful chemicals that become the source of impurities on nanoparticles’ surfaces, limiting their biomedical applications [8].

Comparatively, green synthesis, which involves using plants resources or different microorganisms, is considered a cost-effective and eco-friendly alternative. Green synthesis results in stable and biocompatible nanoparticles [9]. The advantage of green nanoparticles is not only restricted to stability and biocompatibility; in addition, green nanoparticles also exert better antimicrobial action than the chemical or physically synthesized nanoparticles [10]. This enhancement in action is undoubtedly due to biological corona surrounding green nanoparticles, which comes from the source of synthesis [11]. Biological corona comprises various biomolecules, including plants metabolites, flavonoids, carbohydrates, sugar residues, proteins, and amino acids. It reflects the reaction components used in synthesis, which often come from the source [12]. The corona layer also plays a role in the interaction of the nanoparticles in any biological environment, which becomes a potent factor for nanoparticles applications, either to treat pathogen or mammalian cells [13]. In antimicrobial treatment, these corona layers increase in thickness due to the addition of proteins or components released from the pathogens, which helps with the internalization of nanoparticles. In other cases, the corona layer interacts with the pathogens’ membrane and helps nanoparticles deliver their action. Due to the presence of biological corona on green nanoparticles, they show the least toxicity to the animal cells. This helps in various medical applications where humans and animals are the host and multi-drug resistant bacteria are the target pathogens, such as wound or infection treatment, sensors development. Some environmental applications are also very dependent on this property, in which there is a high chance that the human body or cells can come into contact with nanoparticles, for instance, water purification or treatment for disinfectant purposes or packaging. Thus, the presence of biological corona on green nanoparticles is always beneficial in terms of enhancing nanoparticles’ long-term stability and biocompatibility [14]. Despite many advantages, bacterial-mediated synthesis has a few limitations, such as time-consuming purification methods and a lack of understanding of the mechanism of synthesis. Controlling the nanoparticles shape, size, and monodispersity in the solution phase is also a concern [15]. Nevertheless, the advantages of green nanoparticles outweigh these limitations, and researchers continue exploring the potential of cell factories to synthesize green nanoparticles [16].

**Figure 1:**
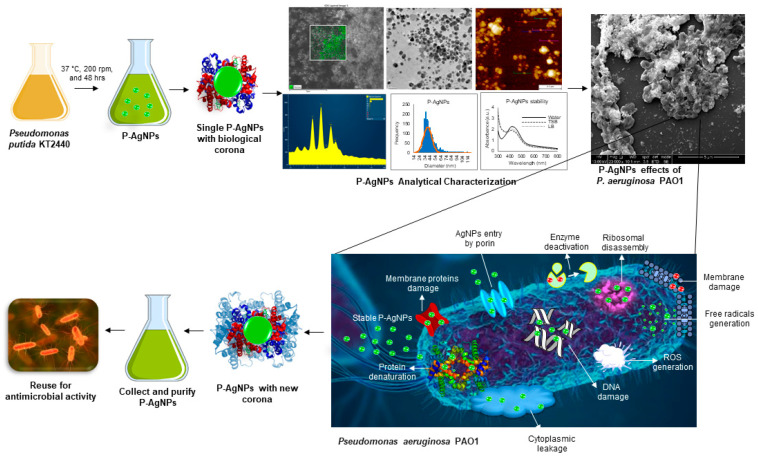
Schematic presentation of P-AgNPs formation from *P. putida* and their antimicrobial activity against *P. aeruginosa* with different mechanisms of action (such as membrane leakage, DNA damage, free radicals generation, reactive oxygen species generation (ROS), protein denaturation, ribosomal disassembly etc.). The figure also describe the hypothesis of corona layer addition and enhancement in antimicrobial activity of nanoparticles, if the host and target organism are closely related.

The current study demonstrates the bacterial synthesis of AgNPs from two common Gram-negative bacteria: *Pseudomonas putida* KT2440 and *Escherichia coli* K12 MG1655. Moreover, the study reports the use of these nanoparticles against human pathogens: *Pseudomonas aeruginosa* PAO1 and *E. coli* UTI 89, which form biofilms underlying chronic infections Figure 1. Our findings indicate that AgNPs are extremely effective when applied to a pathogen that is closely related to the microorganism used for nanoparticle synthesis. This observation has the potential to streamline and facilitate the use of green nanoparticles for more effective antibacterial applications.

## 2. Materials and Methods

### 2.1. Materials

Silver nitrate (AgNO_3_), tryptic soya agar (TSA), and tryptic soya broth (TSB) were purchased from Sigma-Aldrich Chemicals, St. Louis, MO, USA.

### 2.2. Green Synthesis of Silver Nanoparticles

The isolated strain was cultured overnight in 100 mL of TSB at 37 °C, 120 rpm. Next, the growth medium was centrifuged to separate the cells at 8000 rpm for 10 min. The cell-free supernatant was supplemented with 1 mM AgNO_3_ and incubated in a shake flask incubator at 37 °C, 200 rpm, and 24–48 h [17]. The silver salt mix supernatant (reaction medium) was monitored continuously for AgNPs production, by visual inspection, and by recording the UV-Vis spectra of the reaction medium at definite time intervals. To purify the formed nanoparticles, removal of any residues was carried out by centrifugation of the reaction medium at 3000 rpm for 5 min. Then the same medium was centrifuged at 14,000 rpm for 15 min [18]. The supernatant was decanted off to collect the pellets and then washed several times with distilled water. This residue was suspended again into sterile water and used for all experiments.

### 2.3. Analytical Characterization of Nanoparticles

UV-Vis, SEM, TEM, AFM, DLS, FTIR, ICPMS, and TGA studies were done as reported before [19]. For UV-Vis, SEM, TEM, AFM, DLS and ICPMS, aqueous solutions of nanoparticles were used. For FTIR and TGA analysis, purified nanoparticles were air-dried and used in dry form for analysis.

### 2.4. Antimicrobial Activity of P-AgNPs and E-AgNPs

The antimicrobial activity of silver nanoparticles was evaluated against two Gram-negative pathogens: *Escherichia coli* UTI 89, and *Pseudomonas aeruginosa* PAO1. The antimicrobial study was conducted as reported before [10]. Live and dead staining and SEM microscopic observation of dead and control cells were also performed as described before [3].

## 3. Results and Discussion

### 3.1. Biological Synthesis of P-AgNPs and E-AgNPs

**Figure 2:**
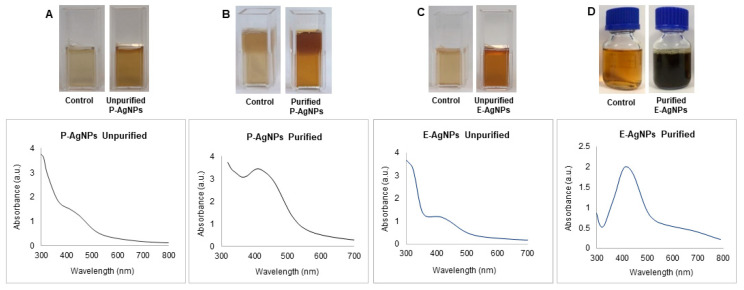
UV-Vis spectra and visible analysis of P-AgNPs and E-AgNPs formation before and after purification of nanoparticles. P-AgNPs unpurified nanoparticles visible and UV-vis spectrum (**A**), purified nanoparticles (**B**). E-AgNPs unpurified nanoparticles visible and UV-vis spectrum (**C**), purified nanoparticles (**D**). Control refer to the visible image of supernatant under similar conditions and without bacteria.

Previously, *P. putida* KT2440 was reported to produce nanoparticles of elemental selenium (nano-Se) from selenite. *E. coli* was also reported to form different metallic nanoparticles, including silver, gold, copper, iron, etc. [20,21]. We incubated the supernatant of 24 h grown culture of *P. putida* and *E. coli* with silver salt for the next 24–48 h. The extracellular supernatant helped in the reduction of the silver salt to nanoparticles. Synthesis of AgNPs occurred extracellularly, which is far more favorable than intracellular synthesis in terms of duration and convenience. With extracellular synthesis, downstream processing processes such as centrifugation and washing procedures, sonication, and other steps can be easily removed. Green synthesis of P-AgNPs and E-AgNPs was first noticed when the color of the synthesis medium changed from pale yellow to deep brown. Origination of brown color in the synthesis medium happened due to the reduction of silver ions to AgNPs. This correlated very well with the known phenomenon of surface plasmon resonance (SPR) property of AgNPs. Images of the original and changed color of the synthesis medium are shown in Figure 2. In addition, we confirmed the synthesis by using UV-Vis spectral analysis. The samples (synthesis media) were scanned from 300–700 nm. UV-Vis spectra for both P-AgNPs and E-AgNPs samples showed maximum absorbance in the 400–500 nm range due to the formation of AgNPs in the medium. Peaks of purified nanoparticles were overlapping in the same region as the original synthesis medium peaks. However, the peaks for the purified medium were sharper and more intense. The AgNPs synthesis occurred extracellularly by *P. putida* and *E. coli*, caused by the microbial enzymes, proteins, or exopolysaccharides (EPSs) such as D-glucose, L-fucose, D-mannose, D-galactose, N-acetyl-D-glucosamine, N-acetyl D-galactosamine released in the synthesis medium [22]. EPS can reduce metal ions to form nanoparticles and act as a capping agent, which helps to stabilize the NPs. As a result, EPSs are being employed to substitute for the microbiological manufacture of various metal nanoparticles [23].

### 3.2. Optimization of Green Synthesis of P-AgNPs and E-AgNPs

**Figure 3:**
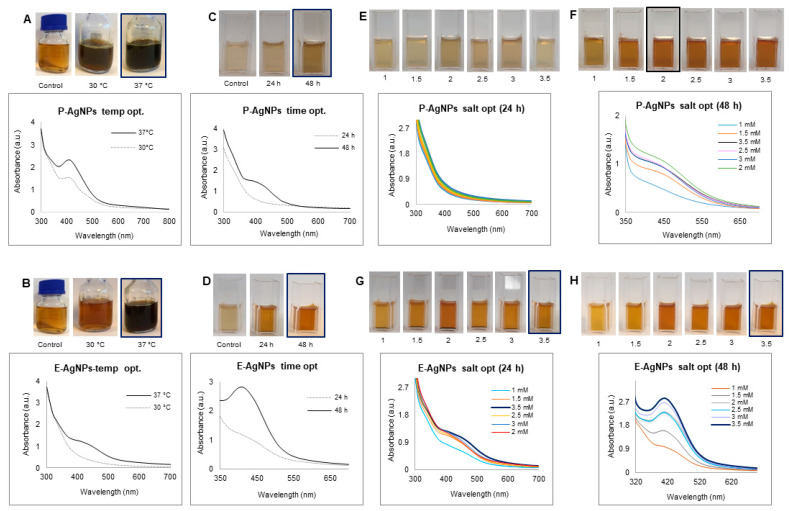
Optimization studies for P-AgNPs and E-AgNPs production. For P-AgNPs, visible picture and UV-Vis spectrum (**A**) temperature optimization, (**C**) time optimization, and (**E**,**F**) silver salt optimization at 24 h and 48 h. For E-AgNPs, visible picture and UV-Vis spectrum (**B**) temperature optimization, (**D**) time optimization, and (**G**,**H**) silver salt optimization at 24 h and 48 h.

Next, we conducted the optimization study for P-AgNPs and E-AgNPs in the respective synthesis media. The results from the temperature optimization study showed that the P-AgNPs formed at both 30 °C and 37 °C; however, the reduction appeared to be more effective at 37 °C, visible as darker color and detectable as a high UV-Vis spectrum peak Figure 3A. Comparatively, for E-AgNPs, reduction was very weak at 30 °C. At 37 °C, the color of the synthesis medium became more blackish-brown, and the spectrum showed the highest absorbance in the 400–500 nm region range. This suggested that for E-AgNPs, the optimum reduction temperature was 37 °C Figure 3B. The time optimization study indicated 48 h as the optimum time needed to reduce silver ions to P-AgNPs and E-AgNPs (Figure 3C,D). In terms of optimizing silver salt concentration, P-AgNPs showed maximum reduction and a sharp peak at 2 mM. E-AgNPs displayed the best results at 3.5 mM. The results of UV-vis spectrum and visible observation were recorded at 24 and 48 h (Figure 3E–H).

### 3.3. Characterization of P-AgNPs and E-AgNPs

**Figure 4:**
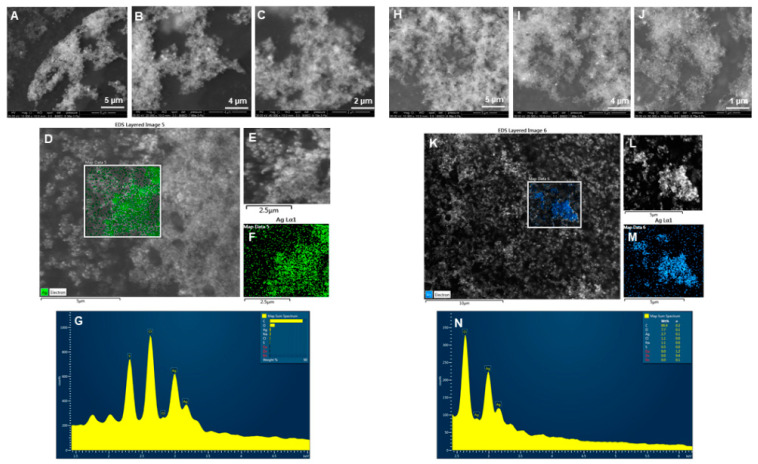
SEM, elemental mapping, and EDX analysis of P-AgNPs and E-AgNPs. For P-AgNPs, (**A**–**C**) SEM images of nanoparticles at different scales, (**D**–**F**) Elemental mapping showing scanned image of NPs with silver element distribution (green), (**G**) EDX spectrum of the elemental mapped region showing sharp peak for silver element. For E-AgNPs, (**H**–**J**) SEM images of nanoparticles at different scales, (**K**–**M**) Elemental mapping showing scanned image of NPs with silver element distribution (blue), (**N**) EDX spectrum of the elemental mapped region showing sharp peak for silver element.

Physicochemical properties determine nanoparticle cellular uptake, transport, and fate for various bio-applications. Considering this, we evaluated some of the most important features of the nanoparticles, primarily the size, stability, and surface chemistry. To analyze the structural morphology, including shape, size, and purity of nanoparticles, we conducted SEM, EDX, elemental mapping, TEM and AFM studies. The SEM images showed the nanoparticles distribution at different scales for P-AgNPs Figure 4A–C and E-AgNPs Figure 4H–J. Elemental mapping reflected the distribution of silver elements for P-AgNPs (Green) Figure 4D–F and E-AgNPs (Blue) Figure 4K–M in the scanned region of SEM micrographs [24]. EDX spectrum was also measured for the respective region, and the sharpest peaks for silver element appeared for both nanoparticles samples Figure 4G,N. A few other surface contaminants appeared, such as carbon and oxygen, which correspond to the carbon tape used for mounting nanoparticles on stab. Na, Cl, S were present in the negligible amount.

**Figure 5:**
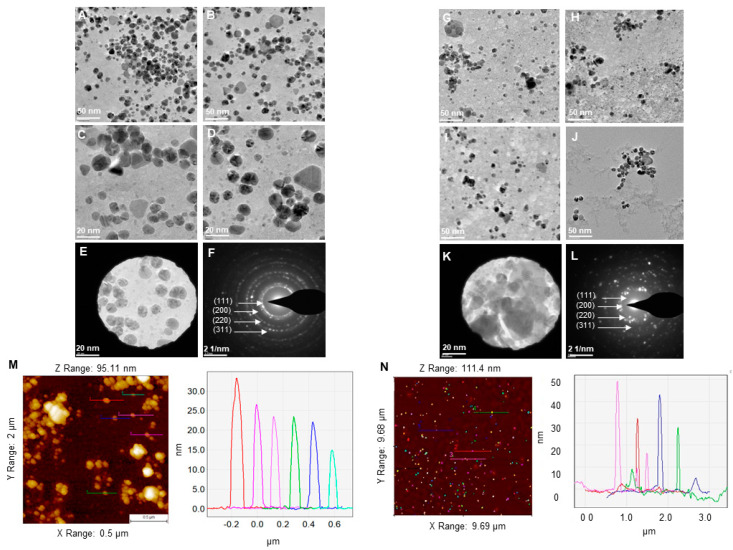
TEM micrographs for (**A**–**D**) P-AgNPs and (**G**–**J**) E-AgNPs. TEM micrograph with visualized SAED aperture and the corresponding SAED pattern for (**E**,**F**) P-AgNPs and (**K**,**L**) E-AgNPs. AFM analysis of (**M**) P-AgNPs and (**N**) E-AgNPs. The colored line on the individual nanoparticles resembles the size of nanoparticles in the respective graph.

According to TEM microscopic images, P-AgNPs showed different morphologies, including spherical, truncated triangle, triangle, and hexagonal Figure 5A–D. The size of P-AgNPs ranged from 15–40 nm. TEM images for E-AgNPs showed that nanoparticles are bigger comparatively and range from 30–70 nm with a polydispersity of spherical, hexagonal, rods, and truncated triangles shape Figure 4G–J. The selected area electron diffraction (SAED) results displayed the crystallinity for both P-AgNPs (Figure 5E,F) and E-AgNPs (Figure 5K,L) by displaying well-defined diffraction rings that correspond to miller indices of (111), (200), (220) and (311), respectively. Despite the slight differences in shape and size of nanoparticles, the crystallinity of the samples showed a strong correlation. AFM showed the height of nanoparticles ranging from 15–30 nm for P-AgNPs (Figure 5M) and 15–50 nm for E-AgNPs (Figure 5N), in accord with the results of TEM analysis [10].

**Figure 6:**
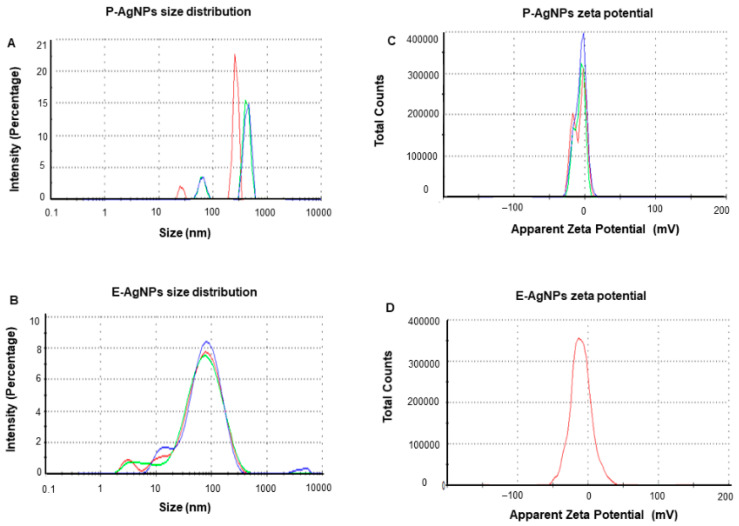
Dynamic light scattering analysis of P-AgNPs and E-AgNPs. (**A**) P-AgNPs distribution concerning size and intensity (**B**) E-AgNPs distribution concerning size and intensity. (**C**) The zeta potential of P-AgNPs and (**D**) E-AgNPs represents a highly negative surface charge. All the measurements were done in triplicates, and the data was calculated as the mean value.

DLS measurements were carried out to determine the average hydrodynamic diameter and the zeta potential of the NPs. The results displayed the size distribution concerning the intensity of nanoparticles with a polydispersity index (PDI), which correlates with their stability. The higher the PDI value is, the less monodispersed the nanoparticles are. For P-AgNPs, the size distribution was 108 nm with PDI 0.732 (Figure 6A), and for E-AgNPs, the size from DLS measured was 47.2 nm with PDI value 0.509 (Figure 6B). Both PDI values appear to be quite small, which means that the nanoparticles were mostly monodisperse. The zeta potential values observed for P-AgNPs and E-AgNPs were −6.12 mV (Figure 6C) and −18.7 mV (Figure 6D), respectively. The negative zeta potential value further confirms the stability of nanoparticles in aqueous solutions [25]. Based on the results, E-AgNPs appear to be more stable than P-AgNPs due to the high negative zeta potential value.

**Figure 7:**
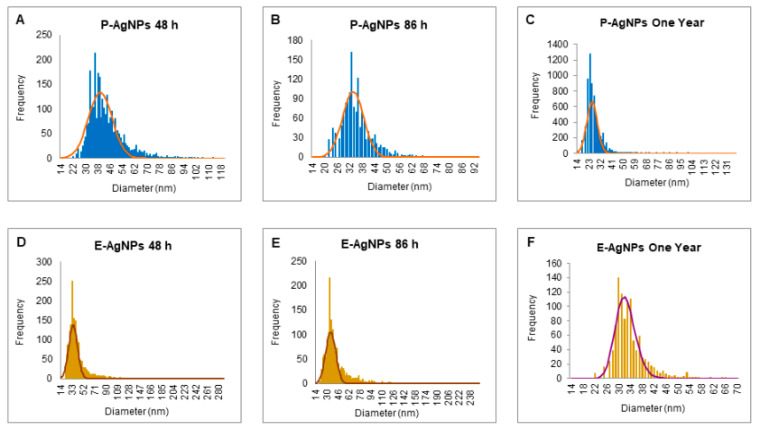
sp-ICPMS and stability analysis of P-AgNPs and E-AgNPs. ICPMS histogram of P-AgNPs at different time intervals, (**A**) after 48 h of synthesis at RT (room temperature), (**B**) after 86 h of synthesis at RT, (**C**) after one year at 4 °C. ICPMS histogram of E-AgNPs at different time intervals, (**D**) after 48 h of synthesis at RT, (**E**) after 86 h of synthesis at RT, (**F**) after one year at 4 °C.

**Figure 8:**
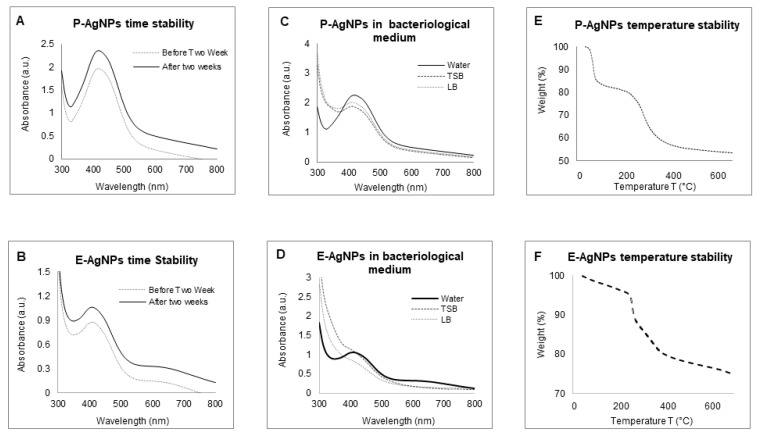
UV-Vis spectrum representing the stability analysis, before and after two weeks of incubation at RT for (**A**) P-AgNPs, and (**B**) E-AgNPs; in a different medium, (**C**) P-AgNPs, (**D**) E-AgNPs; at the temperature range from 20–700 °C measured by TGA instrument, (**E**) E-AgNPs, (**F**) E-AgNPs.

The sp-ICPMS study revealed the concentration of synthesized nanoparticles as 0.108 μg/μL for P-AgNPs and 0.10 μg/μL for E-AgNPs Figure 7. The stability of the nanoparticles as colloids is very important, as unstable nanoparticles will not be able to disperse homogenously, which may affect their antibacterial properties and reduce efficacy [26]. We conducted a stability analysis to investigate the produced NPs stability and aggregation behavior. ICPMS was used to conduct the stability analysis at a difference of two days, three days, and one year. The results showed no variation in the histogram for P-AgNPs (Figure 7A–C) and E-AgNPs (Figure 7D–F), suggesting that the nanoparticles remained stable for a longer period. In addition, time stability analysis was conducted by measuring the UV-Vis of nanoparticles solution in a difference of two weeks kept at room temperatures. The spectrum showed no variation in the P-AgNPs and E-AgNPs samples (Figure 8A,B). A stability test in different bacterial media and water was also conducted. Results displayed that the nanoparticles remain stable in all the dispersion media but give the best peak in water. This could be due to the extra salt, protein, and carbon source present in the medium, which interferes with nanoparticles’ peak (Figure 8C,D). TGA measurements were done to check the temperature stability for nanoparticles. The graph showed that nanoparticles were starting to degrade for temperatures above 200 °C (Figure 8E,F). Further, an increase in temperature causes complete damage to nanoparticles.

**Figure 9:**
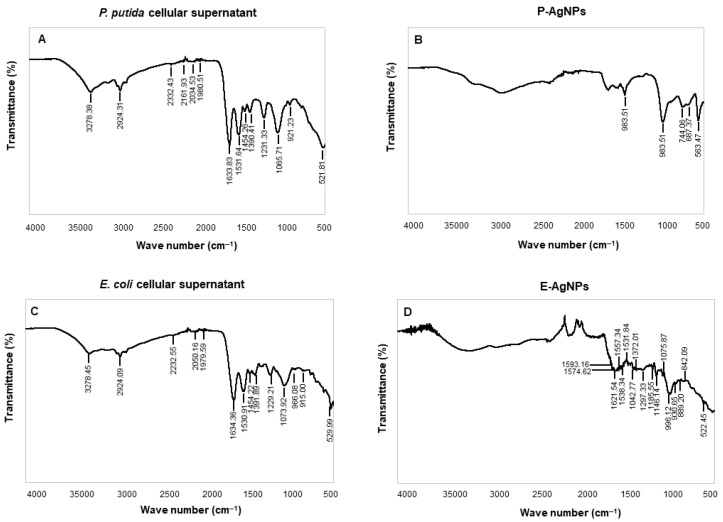
FTIR spectrum of (**A**) freeze-dried cellular supernatant of *P. putida* and (**C**) *E. coli*, and (**B**) pure P-AgNPs, and (**D**) E-AgNPs, which demonstrate the active surface groups for respective samples.

Figure 9 and Table 1 displays the FTIR analysis for biological corona on both the surface of the nanoparticles, i.e., P-AgNPs and E-AgNPs with their respective cellular supernatants. FTIR results for P-AgNPs biological corona confirmed the presence of many active groups: carbonyl −C−O−C or −C−O stretching vibrations of amide linkages, carbohydrates, overlapping of C−O, C−N, C−O−C, C−O−P stretching modes, and C−C deformation. In the case of E-AgNPs, the active groups present in the corona complex were −C=C stretching in the carbonyl group and −C=O stretching vibration of proteins or amide (I). In addition, C−N aromatic amino groups, carbonyl −C−O−C or −C−O stretching vibrations of amide linkages, carbohydrates, overlapping of C−O, C−N, C−O−C and C−O−P stretching modes, and C−C deformation were also present. More surface groups are present on E-AgNPs surface as compared to the P-AgNPs (Table 1); the reason could be the nanoparticles size, as E-AgNPs are bigger in sizes, so provide more surface area for attaching biological residues, since the large surface area of individual nanoparticles provides more surface for interaction with biological moieties. Overall, the FTIR results conclude that it is mostly proteins, amino acids, carbohydrates, and other biological residues such as secondary metabolites, etc., helped in nanoparticles reduction and stabilization [27].

### 3.4. Antibacterial Activity of P-AgNPs and E-AgNPs

**Figure 10:**
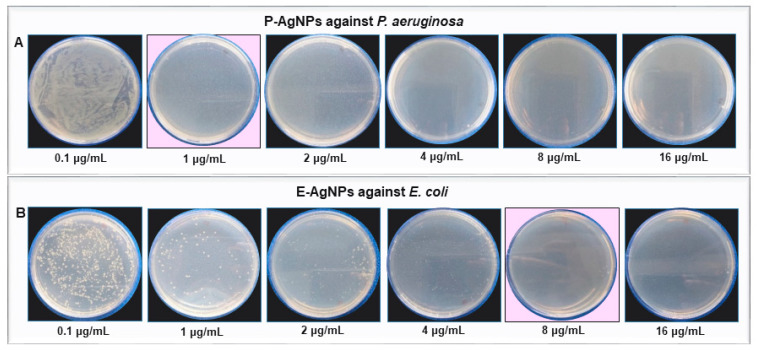
Cell viability test at different concentrations ranging from 0.1–16 µg/mL of (**A**) P-AgNPs against *P. aeruginosa* and (**B**) E-AgNPs against *E. coli*. The pink background shows the MBC values of respective pathogens with complete growth inhibition.

Next, we explored the antibacterial activity of produced P-AgNPs and E-AgNPs against *P. aeruginosa* and *E. coli*, to test the effectiveness and efficiency of nanoparticles against pathogens that are closely related to the bacteria used for nanoparticle synthesis. Figure 10. Our working hypothesis was that the biological corona, which comes from the bacteria used for nanoparticle synthesis, could more effectively interact with the cellular envelope of the targeted pathogen, if they had a similar composition. The antimicrobial activity was first conducted by plate assay, and the results showed that the P-AgNPs completely inhibit bacterial growth for *P. aeruginosa* at 1 µg/mL with no remaining colonies (Figure 10A). E-AgNPs completely inhibited the bacterial growth for *E. coli* at 8 µg/mL (Figure 10B). Both P-AgNPs and E-AgNPs were very effective against *P. aeruginosa* and *E. coli* cells, respectively, and displayed low MBC values. The question was, were they more effective against their respective pathogens than nanoparticles produced in unrelated microbial hosts? There are many reports about green nanoparticles production and their effects against a wide variety of pathogens; however, the concentration of nanoparticles needed to achieve total growth inhibition was always higher in these reports. For instance, Liao et al. displayed the antibacterial activity of chemically synthesized AgNPs against multidrug-resistant *P. aeruginosa* with the MIC range of 1.406–5.625 µg/mL and the MBC range of 2.813–5.625 µg/mL [28]. Chandrasekharan et al. demonstrated the *Gmelina arborea* mediated AgNPs (GA-AgNPs) against *P. aeruginosa* and *E. coli*. The authors showed that the GA-AgNPs exert MIC and MBC value 90 µg/mL against *P. aeruginosa*, and 20 µg/mL of MIC and 40 µg/mL of MBC value against *E. coli* [29]. Comparatively, our P-AgNPs were considerably more effective, with the MBC value at just 1 µg/mL against *P. aeruginosa*. Several factors influence the antimicrobial activity of green nanoparticles, such as size, shape, surface charge, and most importantly, the biological corona that surrounds the nanoparticles. The biological corona forms while synthesizing nanoparticles; however, it modifies while nanoparticles interact in the cellular medium, such as bacteria or mammalian cells [30]. The corona formed around P-AgNPs and E-AgNPs with the help of biological residues present in the supernatant was originated from *P. putida* and *E. coli* cells’ extracellular secretions. In the current study, the targeted pathogen also belonged to the same genus. Our results support the hypothesis that the cellular envelope of similar composition on the target organism allowed the nanoparticles to enter the cells more easily and helped the NPs exert their effects.

**Figure 11:**
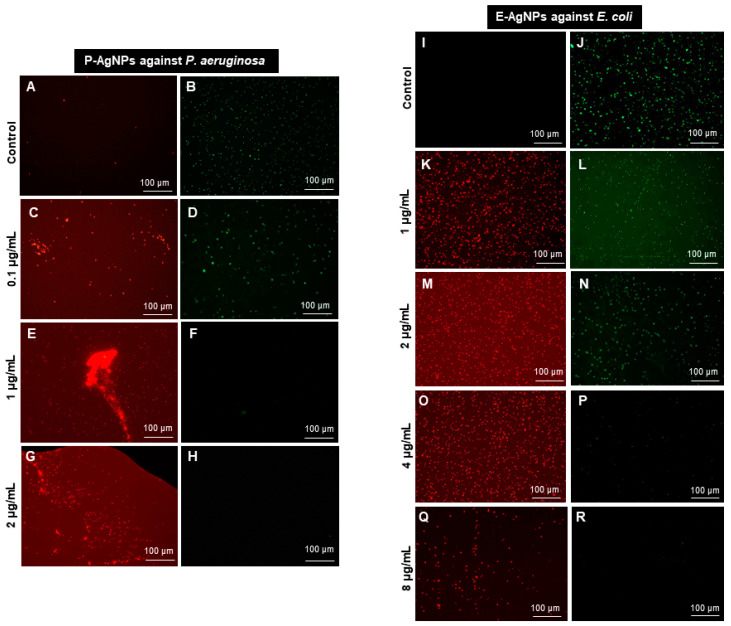
Live and dead staining. (**A**–**H**) P-AgNPs effects on *P. aeruginosa* and (**I**–**R**) E-AgNPs effects on *E. coli*.

We confirmed these results further using the live and dead staining method, where red indicates the dead cells and green represents the live cells [10]. The live and dead assay of nanoparticles against *P. aeruginosa* and *E. coli* were displayed in Figure 11, which aligned with the plate assay results. These pictures show an increasing red region, representing the dead cells, correlated with the nanoparticles concentration. In contrast, the intensity of the green color (live cells) decreases continuously with increasing nanoparticle concentration. Thus, the cells were dying with nanoparticles treatment at a higher dose.

**Figure 12:**
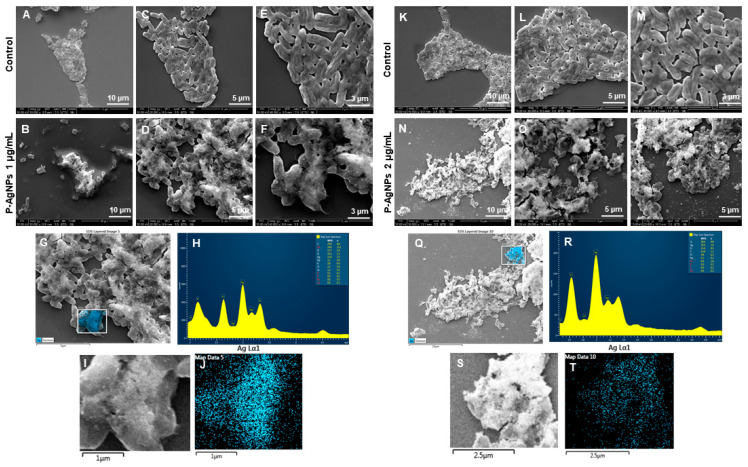
SEM analysis of *P. aeruginosa* cells after treatment with P-AgNPs. (**A**–**E**) Control cells and (**B**–**F**) P-AgNPs treated cells with 1 µg/mL at different scales. (**G**) Scanned image of treated cells, (**H**) EDX spectrum of the chosen area-showing peak for silver element. (**I**,**J**) elemental mapping of the selected area showing silver elements in the treated cells (blue color). (**K**–**M**) Control cells and (**N**–**P**) cells treated with 2 µg/mL of P-AgNPs at different scales. (**Q**) Scanned image of treated cells, (**R**) EDX spectrum of the chosen area showing peak for silver element, (**S**,**T**) elemental mapping of the selected area showing silver elements in the treated cells (blue color).

SEM was further used to reveal the morphological changes of the treated cells. Figure 12 showed the morphology of *P. aeruginosa* treated cells at 1 and 2 µg/mL of P-AgNPs. The cells have entire open structures and porous membranes and are surrounded by nanoparticles on the surface (Figure 12N–P). The image also shows the absence of any slimy covering (biofilm matrix) around the cells, which means nanoparticles inhibited the biofilm formations, which could have protected the cells. On the other hand, the biofilm matrix is clearly present in control untreated cells, which appear to be joined and stick with each other coherently. In the treated pathogens, the damage was caused by the membrane-attached nanoparticles that were able to attach with the membrane easily due to the similar corona composition and caused cell lysis, one of the known mechanisms behind AgNP’s antimicrobial action [31,32]. Another possibility of damage is the easy uptake of nanoparticles by *P. aeruginosa* cells due to the very small size of NPs, spherical shape, and most importantly, similar corona, which allows either endocytosis or engulfment of nanoparticles by treated cells [33]. Once the P-AgNPs are in, there are several mechanisms reported by which nanoparticles cause cell death, such as ROS production, DNA damage, inhibition of proteins and ribosomes functions, etc. [2,16], presented thoroughly in Figure 1. In addition, the negative surface charge on nanoparticles also plays a critical role in causing cytotoxicity. Comparatively, control cells (Figure 12A,C,E,K,L,M) showed the intact cell structure covered under the biofilms and remained safe without losing their identity. The EDX spectrum and elemental mapping were used to scan the treated cells and confirm the presence of nanoparticles covering cell structures. The results showed that the scanned area represented the highest distribution of a silver element in the elemental mapping (blue color), implying that cell lysis was caused by silver element toxicity. EDX spectrum of the mapped region also displayed the highest and sharp peak from silver element without any metallic contamination; thus, a clear sign that the silver is present in the highest amount and causes all the damage in the treated *P. aeruginosa* cells.

**Figure 13:**
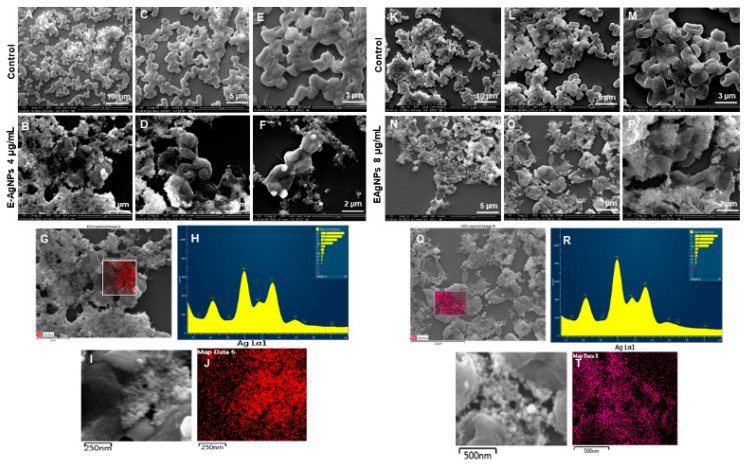
SEM analysis of *E. coli* cells after treatment with E-AgNPs. (**A**–**E**) Control cells and (**B**–**F**) cells treated with 4 µg/mL of E-AgNPs at different scales. (**G**) Scanned image of treated cells, (**H**) EDX spectrum of the chosen area-showing peak for silver element. (**I**,**J**) Elemental mapping of the selected area showing silver elements in the treated cells (red color). (**K**–**M**) Control cells and (**N**–**P**) cells treated with 8 µg/mL of E-AgNPs at different scales. (**Q**) Scanned image of treated cells, (**R**) EDX spectrum of the chosen area showing peak for silver element, (**S**,**T**) elemental mapping of the selected area showing silver elements in the treated cells (pink color).

Figure 13 sho ws the change in structures of *E. coli* cells after E-AgNPs treatment at 4 and 8 µg/mL. The treated cells in Figure 13B,D,E,N,O,P display the cells with shave-off membranes, shrunk outer membranes, or complete loss of cell architecture. Previously, Patra et al. described the antimicrobial activity of green silver nanoparticles originated from an aqueous extract of corn leaf waste of *Zea mays.* The authors demonstrated MIC value of 50 µg/mL and MBC value 100 µg/mL against *E. coli*, which is much higher than the present study [34]. Similarly, Alyousef et al. demonstrated the *Myrtus communis* mediated silver nanoparticles antimicrobial activity with MIC 25 µg/mL and MBC 50 µg/mL values against *E. coli*, in contrast to the current report, which means E-AgNPs produced in this study have great antibacterial activity at a much lower concentration [35]. Similarly to P-AgNPs, in this case, E-AgNPs also seemed to remain attached with the treated pathogen after exerting their effects. A similar mechanism can be predicted for the action of E-AgNPs that might have caused cell lysis is the membrane damage. It would have helped in nanoparticles internalization due to the similar corona layer; nanoparticles, in turn, cause cell organelle damage and cellular leakage. Comparatively, control cells show intact cells without disturbance at different scales. EDX and elemental mapping results indicated that the damage happened due to the presence of silver elements and represent a clear and sharp peak of silver [19].

The interesting feature in the antibacterial activity study is that, after damaging the cells, both P-AgNPs and E-AgNPs do not show any major disturbance in their structure. Figure 12 and Figure 13 show how nanoparticles remain attached in spherical form after cells were lysed completely. The nanoparticles were either released after cell lysis or remained attached to the membrane. In either case, the shape is maintained, reflecting nanoparticles’ stability in a complex environment, which is undoubted because of the biological corona layer around nanoparticles. The literature reports that for any nanomaterials or nanoparticles designed for medicinal purposes, the role of coating agents or corona is very important for surface stabilization and nanoparticles effects. It is a critically important factor in designing and developing safe and efficient nanomaterials [36,37]. Reports also suggest that AgNPs may transform very quickly during their journey through different biological conditions. Depending on the biological environment, they can degrade to an ionic form, cause damage, and reconstruct into a nanoparticulate form [30]. The opportunity here is to isolate the nanoparticles again and reuse them for similar or another purpose. No doubt that the new corona would be different from the previous one, and nanoparticles would act differently. However, the opportunity to retrieve the nanoparticles and reuse them is present, which saves the production cost of nanoparticles and plays a critical role in saving the environment, which often receives damage due to the release of nanoparticles in soil or water (Figure 1).

## 4. Conclusions

The present work was focused on developing P-AgNPs and E-AgNPs from *P. putida* and *E. coli* through physicochemical and biological characterizations. P-AgNPs sized from 15–40 nm and E-AgNPs 30–70 nm were successfully prepared. SEM, TEM, AFM, DLS, ICPMS, FTIR, and stability tests concluded that the nanoparticles are stable, well dispersed, and possess active functional groups. The nanoparticles were further explored for antimicrobial activity against pathogens belonging to the same genus, i.e., *P. aeruginosa* and *E. coli*. The results were evaluated by performing a series of antibacterial tests plate assay, live and dead assay, SEM structural analysis, etc. The antimicrobial activity was evidenced by the change in morphology of treated pathogens at very less concentrations. P-AgNPs completely inhibited the bacterial growth at 1 µg/mL for *P. aeruginosa* and E-AgNPs at 8 µg/mL for *E. coli*. Thus, the AgNPs can show strong antibacterial effects if the nanoparticles are applied in and are produced from closely related microorganisms. This effect is most likely correlated to the composition of the biological corona. In the future, if specific multi-drug resistant bacteria are a target, the preferred strategy we would suggest is to use nanoparticles produced by a closely related microorganism.

## Figures and Tables

**Table 1:** Fourier transform-infrared spectroscopy (FT-IR) spectra for cellular extract of *P. putida*, *E. coli*, P-AgNPs and E-AgNPs.

Type of Bond	Cellular Extract of *P. Putida*	P-AgNPs	Cellular Extract of *E. Coli*	E-AgNPs
−OH (hydroxyl group) of phenolic compounds and N−H group	3278.38		3278.45	
asymmetric stretching of a methyl group −CH_3_ C−H stretching of alkanes or secondary amines	2924.31		2924.09	
Alkyne group	2332.43, 2161.93, 2034.53, 1980.51		2232.55, 2050.16 1979.59	
−C=C stretching in carbonyl group and −C=O stretching vibration of proteins or amide I	1633.83, 1531.64		1634.36, 1530.91	1621.54, 1593.16 1574.62, 1538.34 1557.34, 1531.84
N−H stretching vibration of proteins	1454.26		1454.22	
C−N aromatic amino groups	1390.41, 1231.33		1391.89, 1229.21	1372.01, 1297.33, 1185.55, 1146.14,
Carbonyl −C−O−C or −C−O stretching vibrations of amide linkages, carbohydrates, Overlapping of C−O, C−N, C−O−C and C−O−P stretching modes	1065.71, 921.23	983.51, 983.51	1073.92, 966.08, 915.00	1042.77, 1075.87, 996.12, 930.65, 889.20, 842.09
C−C deformation	521.81	744.06, 687.37, 563.47	529.99	522.45
